# Depressive and Anxiety Symptoms Screening in Cardiac Inpatients: A Virtuous Italian Approach to Psychocardiology

**DOI:** 10.3390/ijerph17145007

**Published:** 2020-07-12

**Authors:** Alessandra Gorini, Mattia Giuliani, Luca Raggio, Simone Barbieri, Elena Tremoli

**Affiliations:** 1 Department of Oncology and Hemato-Oncology, University of Milan, Via Festa del Perdono, 7, 20122 Milan, Italy; 2 IRCCS Centro Cardiologico Monzino, Via Carlo Parea, 4, 20138 Milan, Italy; mattia.giuliani@ccfm.it (M.G.); simone.barbieri@ccfm.it (S.B.); elena.tremoli@ccfm.it (E.T.); 3 Applied Research Division for Cognitive and Psychological Science, European Institute of Oncology IRCCS, 20141 Milan, Italy; luca.raggio@studenti.unimi.it

**Keywords:** cardiovascular diseases (CVD), depressive symptoms, anxiety symptoms, health psychology, screening, PHQ-9, GAD-7

## Abstract

Despite the fact that American Heart Association (AHA) recommended a systematic screening for depression in cardiovascular inpatients, poor attention has been given to this issue. Furthermore, no specific guidelines exist for anxiety screening in cardiovascular inpatients. Thus, the aims of this study were to verify the feasibility of a depressive and anxiety symptoms screening protocol in an Italian hospital specializing in cardiovascular diseases and to evaluate both anxiety and depressive symptoms prevalence. A group of 2009 consecutive inpatients completed the 9-item Patient Health Questionnaire (PHQ-9) and the 7-item Generalized Anxiety Disorder (GAD-7). The rates of depressive and anxiety symptoms were almost 9% and 16% respectively. Men were less likely than women to experience both depressive and anxiety symptoms. Patients who were admitted to the heart failure unit reported higher risk of experiencing both symptoms compared to patients in other wards. Similarly, patients admitted to the cardiac surgery unit showed a higher risk of experiencing anxiety symptoms compared to other patients. The proposed screening procedure showed a good feasibility and acceptance. This study highlighted the importance of implementing a short screening procedure in hospitals dealing with cardiovascular inpatients to identify those individuals who require specific attention and interventions.

## 1. Introduction

Major Depressive Disorder (MDD), commonly referred as depression, is a highly prevalent mental diseases, with more than 300 million people affected worldwide and it has been consistently associated with increased risk of cardiovascular diseases (CVD) [1]. The prevalence of depression in CVD patients is about 15–30% [2], which is two-to-three times more than in the general population. Many studies also indicate that patients diagnosed with depression show a 60% higher risk of developing CVD compared to healthy controls [3]. In patients with CVD, depression is independently associated with poor prognosis and poor health-related quality of life, and disability, recurrent cardiac events, and high mortality [4]. Such findings have been confirmed by a recent European Society of Cardiology (ESC) position paper [5], in which the authors showed that depression following acute coronary syndrome (ACS) significantly increases mortality risk. In the same paper, it has been also shown that patients with comorbid depression and stable coronary heart disease (CHD) demonstrate low adherence to treatments and high resistance to lifestyle changes; they are also unlikely to participate in cardiac rehabilitation programs and they are likely to drop out from them [5]. In 2008, this evidence drove the American Heart Association (AHA) to propose a screening process consisting in a primary administration of the 2-item Patient Health Questionnaire (PHQ-2), eventually followed by a further assessment with the 9-items Patients Health Questionnaire (PHQ-9) and, if necessary, by a psychological assessment [6]. A few years after the publication of the AHA guidelines, Sowden and colleagues conducted a study to evaluate the feasibility of a hospital inpatient’s cardiac unit, finding that it is sufficiently feasible and it does not require intensive resource, to be implemented [4]. Unfortunately, despite the existence of specific recommendations and the evidence supporting their applicability in hospital settings, depression is still often unrecognized and rarely assessed and treated in CVD patients [7].

In addition to depression, anxiety symptoms and anxiety disorders, such as Generalized Anxiety Disorder (GAD) and Panic Disorder (PD), are known to be significantly associated with both CVD onset and progression [7], with the aggravating circumstance that anxiety is even more common than depression being present in more than 50% of patients with heart failure. According to Celano and colleagues, the relationship between anxiety and CVD is a complex and unclear issue and, though no model exists, several hypotheses include both behavioral (i.e., less healthy diet, low physical activity, reduced treatment adherence and more smoking) and physiologic factors (i.e., autonomic, endothelial and platelet dysfunction and inflammation processes) as possible determinants of such comorbidity [7]. Similar to depression, despite all the existing evidences displays the link between CVD and anxiety, no systematic programs have assessed and treated anxiety in inpatients with CVD [8].

Based on the lack of systematic interventions on cardiac patients, the Psychocardiology Team operating at Centro Cardiologico Monzino (CCM), an Italian hospital dedicated to the prevention and treatment of cardiovascular patients, implemented a short screening program based on two validated psychological questionnaires. The two aims of the study included: (1) analyzing the overall feasibility and acceptance of this approach to identify depression and/or anxiety symptoms in inpatients with cardiac illnesses; and (2) identifying the relative prevalence of these two symptoms in such specific cohort of patients.

## 2. Materials and Methods

### 2.1. Sample

The sample initially consisted of 2515 consecutively admitted inpatients to CCM for a cardiac disease. Of the initial sample, 2009 (79.88%) completed the questionnaires, while 506 of them (20.12%) returned them blank or incomplete. The recruitment period lasted 6 months (from January 2019 to July 2019). Institutional Review Board approval was obtained for this study, and all patients provided informed consent before completing the questionnaires.

### 2.2. The Screening Procedure

As part of the hospital admission procedure, each patient received a brief survey about personal and anamnestic data and two validated self-report questionnaires: the 9-item Patient Health Questionnaire (PHQ-9) [9] to assess depressive symptoms and the 7-item Generalized Anxiety Disorder [10] to assess anxiety symptoms. At the end of the survey, the ninth item of the Beck Depression Inventory-II (BDI-II) [11] (i.e., a question related to suicidal ideation) was added. The completed questionnaires were collected daily by a psychologist of the Centro Cardiologico Monzino Psychocardiology Team (CCM-PT), who systematically checked them to determine which patients scored above the cutoff. Those patients whose scores were above the cutoff for depressive and/or anxiety symptoms underwent a clinical assessment with a psychologist of the CCM-PT. Those patients who rated the presence of suicidal risk were immediately visited by a CCM-PT psychologist and, in case of real suicidal ideation, also by a psychiatrist.

### 2.3. Questionnaires

#### 2.3.1. 9-Item Patient Health Questionnaire (PHQ-9)

Depressive symptoms were assessed using the PHQ-9 [9]. PHQ-9 was chosen because of its reliability, simplicity, speed of use, its high correlation with formal interview for MDD diagnosis [9,12] and because it is recommended by the AHA screening guidelines [6]. The PHQ-9 is made of 9 items investigating different depression domains whose scores range from 0 to 3 (total score range: 0–27). According to the AHA guidelines, a PHQ-9 score of 10 should be used as cutoff for depression [6].

#### 2.3.2. 7-Item General Anxiety Disorder (GAD-7)

Anxiety symptoms were assessed using the GAD-7 [10], a questionnaire composed by 7 items, recommended for the screening of anxiety in CVD patients by Conway and colleagues [13]. The total GAD-7 score ranges from 0 to 21, with a cutoff of 8 indicating the presence of significant anxiety symptoms.

#### 2.3.3. Suicidal Risk

At the end of the survey, the ninth item of the Beck Depression Inventory-II [11] was administered in order to evaluate the patient’s suicidal risk. Patients had to choose one option between the following four alternatives: (0) I don’t have any thoughts of killing myself; (1) I have thoughts of killing myself, but I would not carry them out; (2) I would like to kill myself; (3) I would kill myself if I had the chance.

### 2.4. Statistical Analysis

Continuous variables are presented as mean ± standard deviation (SD) if normally distributed or median and interquartile ranges (IQR) if not normally distributed, while categorical variables are presented as absolute numbers and percentages. Chi-square tests were implemented to assess sex difference between groups (under/above cut- off of PHQ-9 or GAD-7). To identify independent predictors of depression or anxiety two logistic regression analysis were performed. All tests were two-sided, and a *p-value* < 0.05 was required for statistical significance. All calculations were computed using the SAS software package (Version 9.4 SAS Institute Inc., Cary, NC, USA).

## 3. Results

### 3.1. Demographic and Clinical Characteristics

The sample primarily consists of men and patients over the age of 60, with a low-medium education. Most of the patients were married and they were admitted to the invasive cardiology or the arrhythmology wards. The median length of hospital stay was 2 days (Table 1).

### 3.2. Psychological Characteristics

Approximately 18% of patients scores were above the cutoff: 9% of them showed anxiety symptoms, 3% depressive symptoms and 6% both anxiety and depressive symptoms (Table 2). Regarding the suicidal ideation, 2% of the patients declared to have experienced it, while another 2% (*n* = 43) did not answer the question.

In general, women showed both higher depressive and anxiety symptoms than men (Table 3). The PHQ-9 score was also positively correlated with the length of hospital stay (r = 0.052; *p* = 0.020), despite the r value is very small.

### 3.3. Anxiety and Depressive Symptoms across the Hospital Wards

Both heart failure and cardiac surgery wards showed the highest row percentage of patients with anxiety and depressive symptoms above the cutoff (Table 4).

Multivariate analysis shows that patients admitted to the heart failure ward have a 2.6 higher risk of presenting depressive symptoms compared to patients admitted in the other wards and that men are 51% less likely than women to experience depressive symptoms. Similar results were found for anxiety symptoms. Patients admitted to the heart failure, but also to the cardiac surgery wards, have respectively a 1.8 and a 1.4 higher risk of presenting anxiety symptoms compared to the patients admitted in the other wards. Moreover, men were found to be 42% less likely than women to experience anxiety symptoms. Finally, education acts as protective factor for anxiety, as shown by the fact that higher educated patients experience less anxiety symptoms (−21% for each unit) compared to the others. All the results are showed in Table 5.

## 4. Discussion

The present study represents the first attempt to perform a systematic psychological screening of all patients consecutively admitted during a 6-month period in an Italian hospital specialized in cardiac diseases. Starting from the increasing evidence of a link between CVDs, depression and anxiety, we have implemented a screening procedure, partially based on the AHA guidelines, that allowed us to investigate both depression and anxiety in cardiac inpatients in a simple and quick way. The screening was based on the administration of PHQ-9 and GAD-7, which are two validated self-report questionnaires widely used in cardiovascular literature. The assessment was completed with a specific question about the presence of suicidal thoughts, extracted from the BDI-II questionnaire. All the patients who obtained a score above the cutoff for depression, anxiety or both received a further psychological assessment performed by a clinical psychologist, followed, when necessary, by a number of personalized support sessions. Moreover, those patients who positively answered the suicidal ideation question were immediately visited by a clinical psychologist for an effective suicidal risk evaluation.

### 4.1. Feasibility and Acceptance

One of the main aims of this study was to evaluate the screening procedure feasibility and acceptance. Regarding this issue, we observed that almost 80% of patients completed the questionnaires, spending on average 5 to 10 min completing them. The need of evaluating its feasibility and acceptance came from the fact that our procedure is, for two reasons, partially different from the procedures proposed by the AHA. First, we could not use the PHQ-2 as first step of the screening because it is not validated in Italian. Second, we added a screening procedure for anxiety that was not included in the AHA guidelines. Despite these changes, our data suggest that the implemented screening procedure was feasible, effective, and well-accepted by the majority of patients. Nevertheless, to really integrate it in the everyday clinical practice, we recommend that patients receive and complete the questionnaires once they are admitted in the hospital wards as part of their routine clinical assessment, instead of during the acceptance phase. In our opinion, these changes will further increase the patients’ adherence rate to the survey, as well as the physicians and nurses’ involvement in the screening process itself, giving them the concrete opportunity to use the collected psychological data to provide a more personalized treatment to the patients, taking into account their clinical condition, and their psychological status [14].

### 4.2. Depressive and Anxiety Symptoms Prevalence

Since, to our knowledge, no other studies have systematically investigated the prevalence of both depression and anxiety symptoms in cardiovascular diseases in Italy. Therefore, we can only compare our data with the data obtained from the general Italian population and from those available in the international literature. Compared to the general Italian population, our sample of CVD patients shows a prevalence of depressive and anxiety symptoms (i.e., almost 9% and 16% respectively) that is, respectively, three and four times higher. These data are in line with the majority of the results obtained in other countries, even if both the methodological (i.e., the sample size differences among studies, the use of different screening tools each with a specific cutoff) and the clinical (i.e., the type of cardiac diseases evaluated) differences among the different studies, as well as the heterogeneity in the patients’ recruitment timing (i.e., during the pre- or post-operative period) and in the patients’ previous psychopathological and cardiac history, may significantly affects anxiety and depressive symptoms bringing to heterogeneous data about their effective prevalence [15]. To reduce this heterogeneity, a shared and unique strategy to collect psychological data from CVD patients should be implemented. This strategy would help both cardiologists and psychologists to better identify the incidence of psychological symptoms across CVD patients and to develop new synergies to better understand the link between heart and mind.

### 4.3. Depressive and Anxiety Symptoms Prevalence in the Hospital Wards

We found significant differences in the prevalence of psychological symptoms among different clinical wards. In particular, we observed that patients admitted to the heart failure ward showed a higher risk of presenting both depressive (i.e., 2.5 higher) and anxiety (i.e., 1.8 higher) symptoms compared to those patients admitted in the other wards. This result is not surprising if we consider that heart failure is frequently associated with poor physical functioning and with an impaired health-related quality of life, as well as with frequent hospitalizations and a high prevalence of depressive and anxiety symptoms in non-hospitalized patients [16]. In addition, patients with heart failure show a high rate of recurrence, leading to frequent chronic condition (i.e., Chronic Heart Failure, CHF) characterized by multiple episodes that can lead to an increase of depressive and anxiety symptoms [16]. Patients admitted to the cardiac surgery ward also showed a higher risk of presenting anxiety symptoms (i.e., 1.4 times higher) compared to the other patients. This result is in line with previous studies showing a high prevalence of anxiety symptoms in the preoperative phase of cardiac surgery [17,18,19,20] mainly caused by the fear and uncertainty related to the surgical procedure [19]. Even if these results can be easily explained by the presence of a certain diagnosis (i.e., heart failure) or condition (i.e., pre-operative status) such data may be very useful to predict the hospital wards or services that need psychological support and interventions.

### 4.4. Depressive Symptoms and Length of Hospital Stay

Another interesting result is that the severity of depressive symptoms, but not of anxiety symptoms, positively correlates with the time spent in the hospital (i.e., length of hospital stay, LHS). Previous studies about this issue were specifically focused on the link between preoperative depressive symptoms and the length of hospital stay showing heterogeneous results: some of them found a significant link between the two [21,22,23], whereas some other studies did not find any association [24]. Nevertheless, our sample is not entirely composed of preoperative patients, so our results involve even other clinical conditions and could be explained perhaps by the fact that pre-existing depressive symptomatology may put a strain on the patient’s physical and mental resources leading to a slower recovery time. Again, these data underline the need of activating specific psychological interventions for depressed inpatients with the primary aim of improving their well-being and the secondary aim of reducing the length of hospital stay with possible positive effects on the health care costs.

### 4.5. Depressive and Anxiety Symptoms in Men and Women

We were also interested in understanding if the prevalence of depressive and anxiety symptoms were different between men and women even in the context of cardiovascular diseases. In line with previous data about the incidence of these psychological symptoms in the general population, and with a recent paper of Buckland and colleagues [25], we found that women with cardiovascular diseases are more likely than men with cardiovascular diseases of manifesting anxiety and depressive symptoms [26].

### 4.6. Education as Protective Factor against Anxiety Symptoms

Finally, a very interesting issue arising from our data regards the role of education as protective factor against anxiety symptoms. This result may be explained by the fact that education gives the possibility to enhance access to health-related information that may help patients to better cope with their illnesses and treatments and to increase their empowerment. This explanation was also discussed in the study conducted by Bjelland and colleagues, in which the authors stated that high educational level seems to protect against both anxiety and depression [27].

### 4.7. Limitations

The main limitation of this study is the heterogeneity of the sample. The two aims of the study included: (1) analyzing the feasibility of the proposed screening method in the population of CVD patients admitted in the hospital and (2) identifying the prevalence of depressive and anxiety symptoms on the entire sample for a period of 6 consecutive months. To achieve these aims, we decided to include all the patients regardless of their pre-existing psychopathological conditions and their past clinical history. Moreover, the adopted data collection did not allow us to collect very specific information about their actual diagnosis. Nevertheless, this approach allowed us to verify the feasibility of applying a screening procedure for depression and anxiety in a real clinical context obtaining very satisfactory results in terms of adherence and knowledge and opening concrete possibilities to create new synergies between the field of cardiology and psychology.

## 5. Conclusions

Our data confirm the increased prevalence of depression and anxiety symptoms in CVD patients supporting the urgent need of developing new strategies to further improve the screening procedures in the cardiovascular patient care process. In particular, we argue that 5 issues are of primary importance. The first one is to implement structured screening procedures also for anxiety symptoms and not only for depressive ones. The second one is to include psychological screening procedures for CVD patients in the hospital routine. The third one is to make both physicians and nurses more aware of the frequent cardiovascular and psychological comorbidity and to give them a directs access to the patient’s psychological data. The fourth one is to specifically monitor the hospital wards that are at most risk of having patients with psychological disorders and to develop psychological support interventions within them. The last one is to offer specific support to inpatient women affected by CVD, who are more susceptible than men to psychological problems such as anxiety and depression. In conclusion, we believe it is fundamental to implement a virtuous model in hospitals with cardiovascular wards where psychologists work together with physicians to provide patients with real bio-psycho-social support, which, from assessment to treatment, could improve the patient’s treatment experience and adherence, quality of life, and reduce the hospitalization time and the related costs.

## Figures and Tables

**Table 1:** Demographic and clinical characteristics of the sample.

		Mean/Median	SD/IQR ^1^	*N*
**Age (mean; SD)**		64.0	14.6	2006
**Education (mean; SD)**		11.6	4.4	1746
**Length of Hospital Stay (median; IQR)**		2	1–4	1973
		**Frequency**	**Percentage**	***N***
**Sex**	Men	1426	70.98	2009
	Women	583	29.02	
**Marital** **Status**	Single	204	10.26	1989
	Unmarried Couples	91	4.58	
	Married Couples	1366	68.68	
	Divorced	149	7.49	
	Widowed	179	9.0	
**Hospital** **Ward**	Heart Failure	232	11.61	1998
	Clinical Cardiology	88	4.4	
	Cardiac Surgery	323	16.17	
	Arrhythmology	632	31.63	
	Invasive Cardiology	723	36.19	

^1^ SD, Standard Deviation; IQR, Interquartile Range.

**Table 2:** Psychological characteristics of the sample.

		GAD-7 ^1^		
		Under Cutoff	Above Cutoff	Total
**PHQ-9 ^2^**	*Under Cutoff*	1642 (81.8%)	189 (9.4%)	1831 (91.1%)
	*Above Cutoff*	53 (2.6%)	125 (6.2%)	178 (8.9%)
	*Total*	1695 (84.4%)	314 (15.6%)	2009
			**Frequency**	**Percentage**
**Suicidal** **Ideation ^3^**		I don’t have any thoughts of killing myself	1926	98.0
		I have thoughts of killing myself, but I would not carry them out	33	1.7
		I would like to kill myself	6	0.3
		I would like to kill myself if I had the chance	1	0.1

^1^ GAD-7, 7-item General Anxiety Disorder; ^2^ PHQ-9, 9-item Patients Health Questionnaire; ^3^ Suicidal Ideation, 9th item of the Beck Depression Inventory-II (BDI-II).

**Table 3:** Sex and psychological characteristics of the sample.

		Men		Women		*p-Value* ^3^
		Frequency	Percentage	Frequency	Percentage	
**GAD-7 ^1^**	*Under Cutoff*	1239	86.9	456	78.22	<0.001
	*Above Cutoff*	187	13.1	127	21.8	
**PHQ-9 ^2^**	*Under Cutoff*	1329	93.2	502	86.1	<0.001
	*Above Cutoff*	97	6.8	81	13.9	

^1^ GAD-7, 7-item General Anxiety Disorder; ^2^ PHQ-9, 9-item Patients Health Questionnaire; ^3^ The *p-values* are related to Chi-square test.

**Table 4:** Depressive and anxiety symptoms in the different hospital wards.

		PHQ-9 ^1^		GAD-7 ^2^	
		Under Cutoff	Above Cutoff	Under Cutoff	Above Cutoff
		*N (row %)*	*N (row %)*	*N (row %)*	*N (row %)*
**Hospital** **Wards**	Heart Failure	194 (83.62)	38 (16.38)	181 (78.02)	51 (21.98)
	Clinical Cardiology	81 (92.05)	7 (7.95)	76 (86.36)	12 (13.64)
	Cardiac Surgery	290 (89.78)	33 (10.22)	261 (80.80)	62 (19.20)
	Arrhythmology	587 (92.88)	45 (7.12)	542 (85.76)	90 (14.24)
	Invasive Cardiology	668 (92.39)	55 (7.61)	625 (86.45)	98 (13.55)

^1^ PHQ-9, 9-item Patients Health Questionnaire; ^2^ GAD-7, 7-item General Anxiety Disorder.

**Table 5:** Multivariate analysis: Risk factors for both anxiety and depressive symptoms.

		PHQ-9 ^1^		GAD-7 ^2^	
		OR	95% CI	OR	95%CI
**Age**		1.01	1.000–1.206	1.00	0.986–1.006
**Sex (women)**		0.49	0.340–0.700 *	0.58	0.434–0.770 *
**Education**		0.92	0.765–1.110	0.79	0.677–0.913 *
**Marital Status ^3^**	Single and Divorced	1.24	0.771–1.981	0.93	0.638–1.365
	Widowed	1.25	0.710–2.212	0.80	0.483–1.341
**Hospital Wards ^4^**	Cardiac Surgery	1.42	0.900–2.230	1.43	1.005–2.029 *
	Heart Failure	2.59	1.678–4.000 *	1.83	1.250–2.665 *

^1^ PHQ-9, 9-item Patients Health Questionnaire; ^2^ GAD-7, 7-item General Anxiety Disorder; ^3^ The Marital Status reference category is Couples, which includes both Unmarried and Married Couples; ^4^ The Hospital Wards reference category is *Other Wards*, which includes Clinical Cardiology, Arrhythmology and Invasive Cardiology. * boldface indicates a *p* < 0.05.
